# Different Impacts of Long-Term Tillage and Manure on Yield and N Use Efficiency, Soil Fertility, and Fungal Community in Rainfed Wheat in Loess Plateau

**DOI:** 10.3390/plants13243477

**Published:** 2024-12-12

**Authors:** Mengni Chen, Hailiang Yang, Qingshan Yang, Yongshan Li, Hui Wang, Juanling Wang, Qiaolan Fan, Na Yang, Ke Wang, Jiancheng Zhang, Jiawei Yuan, Peng Dong, Lu Wang

**Affiliations:** 1 Cotton Research Institute, Shanxi Agricultural University, Yuncheng 044000, China; 2 State Key Laboratory of Sustainable Dryland Agriculture (in Preparation), Shanxi Agricultural University, Taiyuan 030031, China; 3 College of Agriculture, Shanxi Agricultural University, Taigu 030801, China

**Keywords:** no-tillage, manure, soil fertility, soil fungal community diversity, NUE, high-throughput sequencing, rainfed wheat field

## Abstract

Conservation tillage and fertilization are widely adopted in agricultural systems to enhance soil fertility and influence fungal communities, thereby improving agroecosystems. However, the effects of no-tillage combined with manure on grain yield, nitrogen use efficiency (NUE), soil fertility, and rhizosphere fungal communities remain poorly understood, particularly in rainfed wheat fields on the Loess Plateau. A 15-year field experiment was conducted at the Niujiawa Experimental Farm of the Cotton Research Institute, Shanxi Agricultural University. Five treatments were assessed: conventional tillage without fertilizer (C), no-tillage with chemical fertilizer (NT), no-tillage with chemical fertilizer and manure (NTM), conventional tillage with chemical fertilizer (T), and conventional tillage with chemical fertilizer and manure (TM). The results demonstrated that the NTM treatment significantly increased grain yield by 124.95%, NT by 65.88%, TM by 68.97%, and T by 41.75%, compared to the C treatment (*p* < 0.05). NUE in the NTM treatment was improved by 58.73%–200.59%. Compared with the C treatment, NTM significantly enhanced soil nutrients, including organic matter (OM) by 70.68%, total nitrogen (TN) by 8.81%, total phosphorus (TP) by 211.53%, available nitrogen (AN) by 90.00%, available phosphorus (AP) by 769.12%, and available potassium (AK) by 89.01%. Additionally, the NTM treatment altered the rhizosphere fungal community of winter wheat, with *Ascomycota* (81.36%–90.24%) being the dominant phylum, followed by *Mucoromycota* (5.40%–12.83%) and *Basidiomycota* (1.50%–8.53%). At the genus level, NTM significantly increased the abundance of *Mortierella* and *Dendrostilbella*. An α-diversity analysis revealed that the richness and diversity of soil fungi were highest under NTM. The unweighted pair-group method with arithmetic mean (UPGMA) and principal coordinates analysis (PCoA) based on Bray-Curtis distances indicated that NTM formed a distinct fungal community with the highest phylogenetic diversity, which differed significantly from other treatments. Redundancy analysis (RDA) demonstrated that soil chemical properties variably influenced fungal community dynamics, with higher abundances of *Ascomycota* and *Zoopagomycota* positively correlated with OM, AN, AP, TP, and AK. Correlation analysis showed that wheat yield and NUE were positively correlated with *Mortierella* and *Dendrostilbella*, and negatively correlated with *Fusarium*, *Chaetomium*, and *Alternaria*. In conclusion, no-tillage with manure not only enhanced soil fertility but also enhanced soil fungal community structure, leading to greater wheat yield and NUE. These findings provide guidance for agricultural practices in rainfed wheat fields of the Loess Plateau.

## 1. Introduction

Tillage and fertilization are critical agricultural practices that directly or indirectly impact the physical, chemical, and biological properties of soils, thereby influencing soil fertility and crop productivity [1,2]. Conventional tillage (e.g., plow tillage) has been associated with reduction of agroecosystem health and global food security [3]. In response, no-tillage (NT) has gained popularity worldwide as a conservation practice [4]. NT can conserve soil moisture, alter soil properties [5], and enhance both the grain yield of winter wheat and nitrogen use efficiency (NUE) [6]. However, studies indicate that the benefits of NT on crop yield may vary depending on aridity, fertilization practices, and soil fertility [7,8].

With a growing global population and increasing food demand, excessive chemical fertilizer use for maximizing yields has led to adverse effects, such as soil quality degradation, reduced nutrient use efficiency, elevated greenhouse gas emissions, and decreased crop productivity. In China, the average nitrogen agronomic efficiency (NAE) for wheat was 9.2 kg/kg between 2000 and 2011, 36% lower than the global average of 14.3 kg/kg [9,10]. Furthermore, nitrogen recovery efficiency for major crops in China, such as rice, wheat, and maize, was limited to 28.3%, 28.2%, and 26.1%, respectively [11]. Integrating organic sources has been shown to enhance the efficiency of inorganic fertilizers and improve soil properties [12,13,14]. However, the long-term impacts of NT combined with manure on wheat yield and nitrogen utilization remain under-explored.

Fungi are key contributors to organic matter decomposition and nutrient cycling in agricultural systems. As sensitive indicators of soil quality, fungal communities rapidly respond to environmental changes [15]. Soil fungal community structures are significantly influenced by agricultural practices, which affect the soil’s physical and chemical characteristics [16,17]. NT is known to preserve soil structure [18], benefit soil microbial communities [19], and enhance soil fertility, which is essential for stable crop yields. Studies have indicated that conventional tillage tends to favor bacterial dominance, while conservation tillage supports fungal prevalence in soil microbial communities [20,21,22,23]. Conservation tillage enhances both fungal and bacterial biomass [24] with NT specifically increasing arbuscular mycorrhizal fungal species richness, evenness, and diversity [25]. Previous research has also highlighted that NT creates a favorable environment with higher soil moisture and moderate temperatures, which promote fungal growth [16,26]. By minimizing soil disturbance, NT preserves the integrity of fungal hyphal networks, leading to greater biomass accumulation under suitable conditions [27]. Nevertheless, some studies have reported that NT has no significant impact of NT on soil fungal diversity [28,29], likely due to differences in soil types or interactions with other agricultural practices, such as straw incorporation. Long-term NT may also negatively impact soil health, favoring saprophytic fungi over beneficial symbiotic fungi.

Fertilization also plays a crucial role in shaping soil fungal communities [30,31]. Both organic and inorganic fertilizers generally increase soil nutrient content and fungal abundance, although they may reduce fungal diversity [32,33,34,35]. Organic fertilizers tend to enhance fungal growth and spore production, mitigate the adverse effects of sole chemical fertilizer use, and increase fungal abundance [36,37,38]. Organic addition provides a carbon and energy source for microorganisms [39,40], which can elevate fungal biomass, diversity, and dominance of key groups [41]. Hence, fertilizers, whether organic or inorganic, can significantly boost soil nutrient levels and fungal diversity.

Tillage and fertilization are widely used agricultural measures that affect the composition and function of soil fungal communities. The effects of tillage or manure as a single factor on fungal communities have been studied extensively in farmland ecosystems. Little is known about their combined effects on soil fungal community and ultimately crop yield. Therefore, the responses of the soil fungal community to long-term no-tillage and manure remain unclear. To clarify the composition and diversity of microbial communities, high-throughput sequencing RNA and DNA technology has been used to provide information about their composition, abundance, and structure [42]. For instance, alpha and beta diversity indicate the similarity and difference of microbial distribution diversity and uniformity among different treatments [43].

In the present study, we compared the performances of different tillage and manure treatments in a long-term field experiment in a typical dryland region of the Loess Plateau in China. This study aimed to understand the combined effects of tillage and manure on yield, nitrogen use efficiency, and the soil fungal community. In particular, we investigated (i) the effects of different tillage and manure on crop yield and nitrogen use efficiency, (ii) the effects of different tillage and manure on the soil fungal diversity and structure, and (iii) the relationships between different soil physiochemical properties under different tillage and manure systems and the changes in the soil fungal community.

## 2. Results

### 2.1. Wheat Yield and N Use Efficiency

One-way analysis of variance (ANOVA) analysis revealed that different tillage and manure treatments significantly affected grain yield and nitrogen use efficiency (NUE) (Table 1). The NTM treatment led to a remarkable increase in grain yield by 124.95%, followed by NT 65.88%, TM 68.97%, and T 41.75% compared to the control (C) (*p* < 0.05). Both no-tillage and manure applications significantly enhanced NUE indicators (AE_N_, PEP_N_, and RE_N_). In the NTM treatment, AE_N_, PEP_N_, and apparent RE_N_ improved by 199.30%, 58.73%, and 200.59%, respectively, compared to the T treatment. Other treatments did not show significant differences in these indicators. The accumulated RE_N_ in the NTM treatment increased significantly by 72.08% compared to that of the T treatment, which was significantly different from the other treatments.

### 2.2. Soil Properties Analysis

Long-term soil tillage and fertilization showed significant impacts on soil properties (Table 2). Soil pH ranged from 8.00 to 8.19, with the lowest value observed in the NTM treatment and the highest in the control (C) (*p* < 0.05). Soil organic matter (OM) followed the order of NTM > TM > NT > T > C, though there were no significant differences among TM, NT, and T (*p* > 0.05). Total nitrogen (TN) levels were higher in NTM, T, and C treatments compared to those of NT and TM (*p* < 0.05), but no significant difference was found between NTM and C (*p* > 0.05). The NTM treatment showed significant enrichment of OM, TN, total phosphorus (TP), available nitrogen (AN), available phosphorus (AP), and available potassium (AK) compared to the control (C) (*p* < 0.05). Additionally, TP, AN, AP, and AK were significantly higher in the manure treatments (TM and NTM) than in the NT and T treatments (*p* < 0.05).

### 2.3. Alpha Diversity of Soil Fungal Community

**Figure 1:**
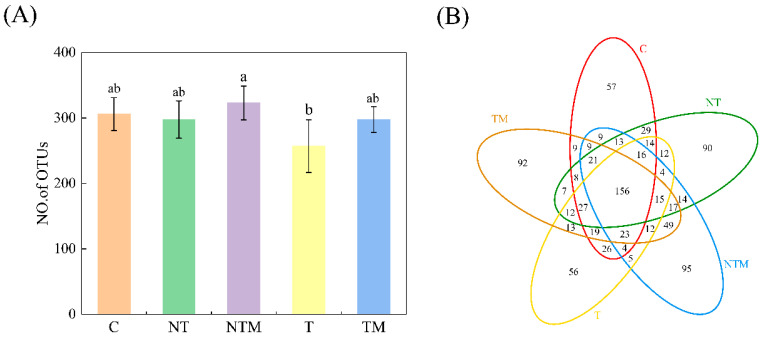
(**A**) The number of OTUs of fungal communities under different treatments. Different lowercase letters indicate significant differences at *p* < 0.05. The error bar represents SD. (**B**) Venn chart based on the operation classification unit (OTU). Each petal corresponds to a sample group. The shared overlapping area represents the OTUs of all samples. The number on a single petal represents the number of OTUs unique to a given sample group.

There were 306, 298, 323, 257, and 298 OTUs (Operational Taxonomic Units) identified in the C, NT, NTM, T, and TM treatments, respectively (Figure 1A). Compared to the T treatment, the number of OTUs was significantly increased by 19.07%, 15.95%, 25.68%, and 15.95% in the C, NT, NTM, and TM treatments, respectively (*p* < 0.05). In manure treatments (NTM and TM), the OTU count was increased by 8.39%–15.95% compared to the chemical fertilizer treatments (NT and T). Likewise, no-tillage treatments (NT and NTM) showed an 8.39%–15.95% increase in OTUs compared to conventional tillage methods. Additionally, 156 OTUs were shared among all treatments, while unique OTUs were observed as follows: 57 in C, 90 in NT, 95 in NTM, 56 in T, and 92 in TM (Figure 1B). The NTM treatment had the highest number of unique OTUs, while the T treatment had the lowest.

Statistically significant differences in alpha diversity were observed among the different tillage treatments based on OTUs (*p* < 0.05; Table 3). The NTM and TM treatments had higher Chao1 index values, while the NT and T showed lower values. No significant differences in the Chao1 index were found among NT, C, and T treatments. For the Shannon index, NTM and NT exhibited higher values, whereas T and TM were lower. There was no significant Shannon index difference between TM and C. The Simpson and ACE indices did not show significant variation among treatments. Evenness was higher in T and NT, while lower in NTM and TM, with no significant differences between TM and C.

### 2.4. Composition of Fungal Community

**Figure 2:**
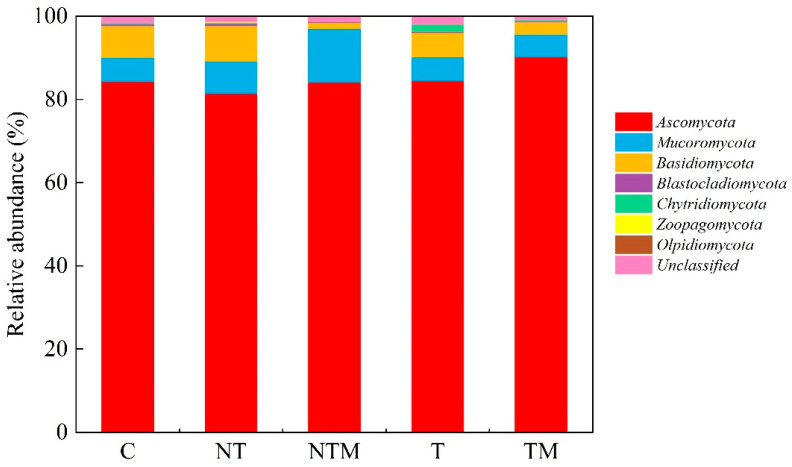
Relative abundances of fungal community under different treatments at phylum level.

**Figure 3:**
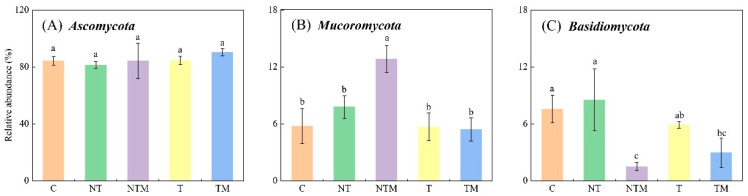
The relative abundance of the main 3 fungi phyla under different treatments. Different lowercase letters indicate significant differences at *p* < 0.05, while the same letters indicate nonsignificant differences at *p* > 0.05. The error bar represents SD.

Across all soil samples, the dominant fungal phyla were *Ascomycota*, *Mucoromycota*, and *Basidiomycota*, which comprised 81.36%–90.24%, 5.40%–12.83%, and 1.50%–8.53% of the fungal community, respectively (Figure 2). The abundance of *Ascomycota* followed the order of TM > T > C > NTM > NT, although the differences among treatments were not statistically significant. In contrast, the abundances of *Mucoromycota* and *Basidiomycota* showed statistically significant differences among treatments (*p* < 0.05) (Figure 3).

**Figure 4:**
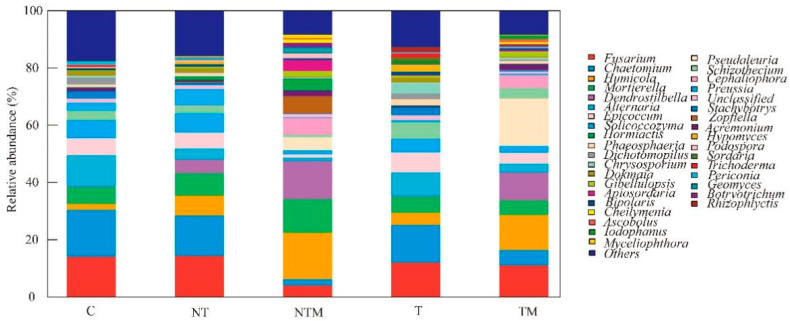
Relative abundances of the fungal community under different treatments at the genus level.

**Figure 5:**
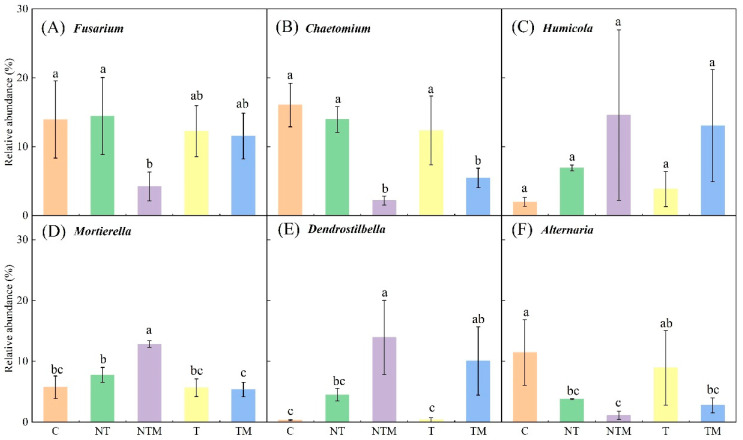
The relative abundance of the 6 main fungi genera under different treatments. Different lowercase letters indicate significant differences at *p* < 0.05, while the same letters indicate nonsignificant differences at *p* > 0.05. The error bar represents SD.

Soil tillage and fertilization regimes significantly altered the composition of the fungal community at the genus level (Figure 4). We examined the six dominant genera—*Fusarium*, *Chaetomium, Humicola, Mortierella, Dendrostilbella,* and *Alternaria* (Figure 5). Notably, the relative abundance of *Fusarium* was lowest under the NTM treatment, which was significantly different from the other treatments (*p* < 0.05). The relative abundances of *Chaetomium* in the NTM and TM treatments also showed significant differences compared to the other three treatments (*p* < 0.05). While *Humicola* exhibited the highest relative abundance in the NTM treatment, no significant differences were observed among other treatments. Conversely, *Mortierella* was significantly more abundant in the NTM treatment than in the other treatments (*p* < 0.05). Similarly, *Dendrostilbella* had the highest relative abundance under NTM treatment, significantly higher than other treatments except for TM (*p* < 0.05). In contrast, *Alternaria* was most abundant in the C treatment, significantly exceeding that of the NTM treatment (*p* < 0.05) (Figure 5). These results indicated that the NTM treatment significantly enhanced the abundance of *Mortierella* and *Dendrostilbella*.

**Figure 6:**
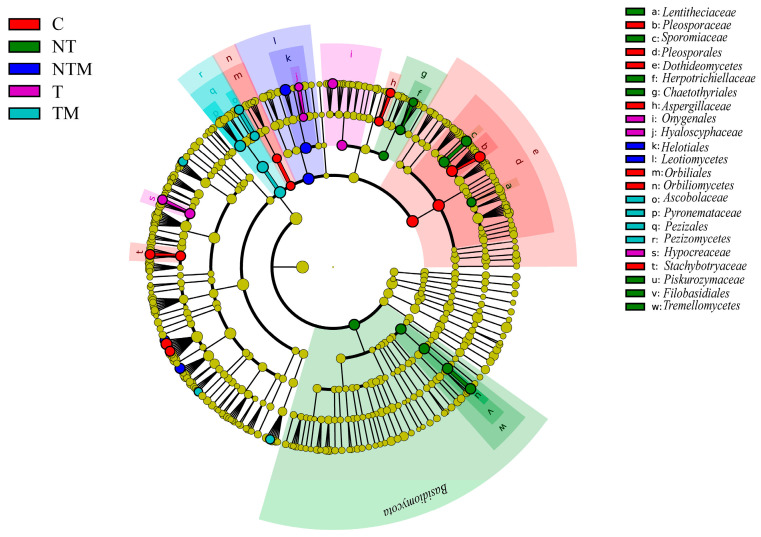
Cladogram based on LEfSe analysis of soil fungal community under different treatments. Color nodes indicate the taxa under different treatments. The diameter of each node shows the relative abundance of each taxon.

We further identified the responses of biomarkers to different tillage and manure treatments by using LEfSe analysis. A total of 42 significant biomarkers were detected within the fungal microbial community (Figure 6). The C treatment had 12 significant biomarkers (2 orders, 2 families, 3 genera, and 5 species), while the NT treatment had 11 significant biomarkers (1 class, 1 order, 2 families, 4 genera, and 3 species). The NTM treatment showed 5 significant biomarkers (1 order, 1 family, and 3 species), whereas the T treatment had 6 significant biomarkers (1 family, 2 genera, and 3 species), and the TM treatment had 8 significant biomarkers (1 order, 1 family, 2 genera, and 4 species). Notably, *Dothideomycetes, Pleosporales, Chaetomium,* and *Alternaria* were significantly enriched in the C treatment. In the NT treatment, *Basidiomycota, Sporormiaceae*, and *Tremellomycetes* were notably enriched, while *Leotiomycetes* and *Helotiales* were significantly enriched in the NTM treatment. Additionally, *Hypocreaceae*, *Chrysosporium*, and *Onygenales* were significantly enriched in the T treatment, and *Pezizales, Pezizomycetes,* and *Pyronemataceae* were notably enriched in the TM treatment. These results indicated that the NT and NTM treatments significantly altered the composition of soil microbial species.

### 2.5. Beta Diversity of Soil Microbial Community

**Figure 7:**
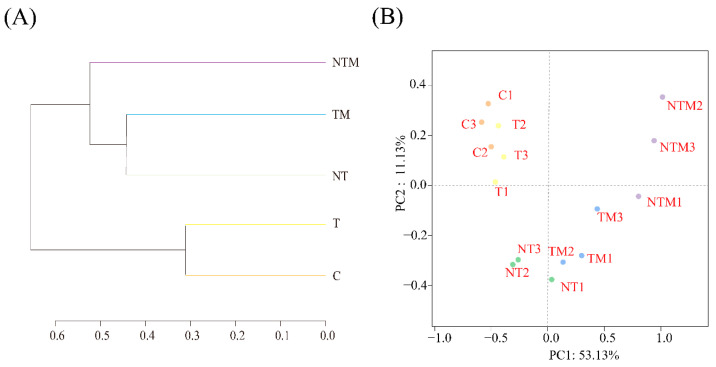
(**A**) Hierarchical clustering tree of fungal community at the OTU level (97% sequence similarity) based on Bray-Curtis distances. (**B**) principal coordinates analysis-PCOA of the fungal community. The PCoA plot was based on Bray-Curtis distances at the OTU level (97% sequence similarity) for the fungal community.

Based on the binary Bray-Curtis distance, Principal Coordinates Analysis (PcoA) was conducted to examine the differences in the rhizosphere fungal community of winter wheat under various tillage and fertilizer treatments (Figure 7). In terms of fungal community composition, the first and second axes accounted for 53.13% and 11.13% of the total variation in fungal community composition, respectively. The C and T treatments, NT and TM treatments, and NTM treatments were distinctly separated in the analysis. Clustering results indicated that the fungal microbial communities in the C and T treatments formed a cohesive group, clearly different from that of the NT, TM, and NTM treatments. Among these groups, *Ascomycota* was the most abundant phylum, followed by *Mucoromycota* and *Basidiomycota*. Notably, *Basidiomycota* was more abundant in the NT treatment, while *Mucoromycota* was more prevalent in the NTM group.

### 2.6. Relationship Between Fungal Community and Soil Properties, Yield, and NUE

**Figure 8:**
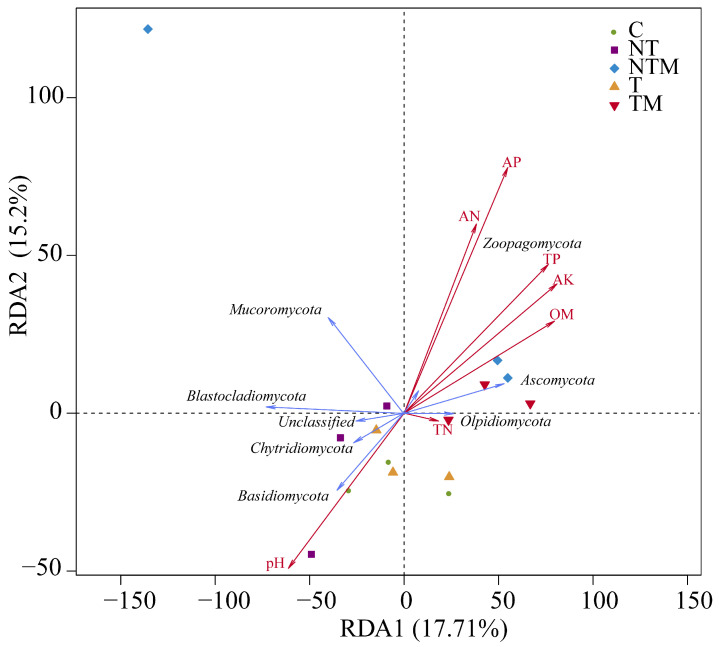
Redundancy analysis (RDA) of the relationship between the distribution of fungal community at phylum level and soil properties.

Redundancy Analysis (RDA) of soil properties and fungal communities indicated that the first ordination axis (RDA1) explained 17.71% of the variation, while the second ordination axis (RDA2) accounted for 15.20% of the variation, and RDA1 and RDA2 together explained a total of 32.91% of the variation (Figure 8). The correlations between dominant fungal communities and soil properties varied. *Ascomycota* and *Zoopagomycota*, which exhibited relatively high abundance, positively correlated with OM, AN, AP, TP, and AK. Conversely, *Basidiomycota* and *Chytridiomycota* showed negative correlations with soil pH, while *Blastocladiomycota* and *Mucoromycota* displayed no significant correlations with soil properties. The composition of the fungal community in different treatments was associated with specific soil properties; in particular, the communities in NTM and TM treatments were positively correlated with *Ascomycota* and *Zoopagomycota*, whereas the communities in C, T, and NT treatments were negatively correlated with Basidiomycota and Chytridiomycota.

**Figure 9:**
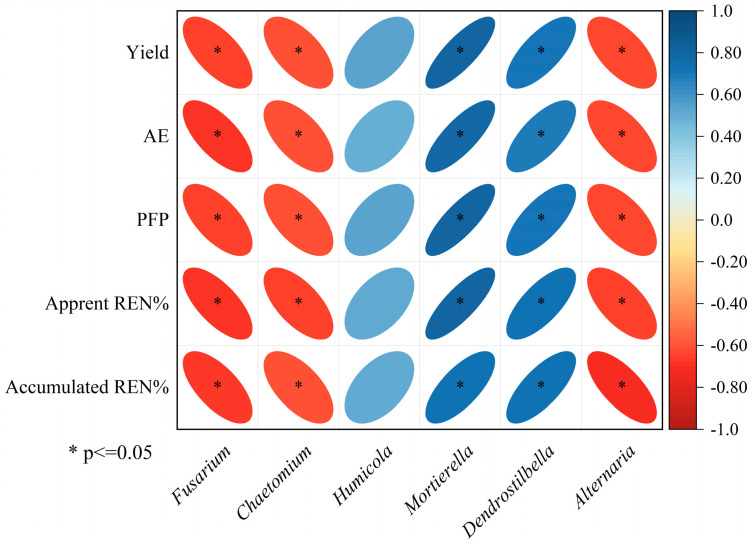
Correlation of fungal community with yield and N use efficiency at genus level.

Additionally, Spearman’s correlation analysis revealed relationships between microbial genera and wheat yield as well as nitrogen use efficiency (NUE). As shown in Figure 9, *Mortierella* and *Dendrostilbella* exhibited significant positive correlations with wheat yield, agronomic efficiency of nitrogen (AE_N_), partial factor productivity of nitrogen (PFP_N_), apparent recovery efficiency of nitrogen (RE_N_), and accumulated REN (*p* < 0.05). In contrast, *Fusarium, Chaetomium,* and *Alternaria* were negatively correlated with these same metrics (*p* < 0.05). *Humicola* showed no significant correlation with wheat yield, AE_N_, PFP_N_, apparent RE_N_, or accumulated RE_N_ (*p* < 0.05).

## 3. Discussion

### 3.1. Effects of Long-Term Soil Tillage and Fertilization Management on Wheat Yield and N Use Efficiency

No-tillage and manure application not only enhanced winter wheat yield but also improved nitrogen use efficiency (NUE) (Table 1). In this study, the no-tillage with manure (NTM) and no-tillage (NT) treatments resulted in yield increases of 33.13% and 17.03%, respectively, compared to conventional tillage systems (TM and T). Additionally, compared to TM and T, the apparent nitrogen use efficiency indicators of the NT and NTM treatments demonstrated significant improvements: agronomic efficiency of nitrogen (AE_N_) increased from 57.80% to 81.24%, partial factor productivity of nitrogen (PFP_N_) increased from 17.04% to 33.09%, apparent recovery efficiency of nitrogen (RE_N_) increased from 55.14% to 80.43%, and accumulated RE_N_ increased from 25.64% to 28.96%. The higher N use efficiency of NTM and NT was primarily due to higher wheat yield. This was consistent with the study of Ding et al. [6]. There are reports that NT has greater soil water storage [44], higher soil available N and root uptake, and superior wheat growth and yield [6]. When compared to NT and T, the application of manure (NTM and TM) resulted in yield increases of 35.60% and 19.20%, respectively. Dutta et al. [5] also indicated that the combination of no-tillage and nutrient management led to increased wheat yields. In particular, the NTM treatment significantly increased wheat yield by 58.70%, AE_N_ by 199.27%, PFP_N_ by 58.73%, apparent RE_N_ by 200.59%, and accumulated RE_N_ by 72.08% compared to conventional tillage (T). Therefore, it is imperative to integrate no-tillage practices with optimal nutrient management strategies to achieve higher wheat yields and improve NUE.

### 3.2. Effects of Long-Term Tillage and Fertilization Management on Soil Properties

Long-term tillage and fertilization management significantly influence soil properties. As shown in Table 2, notable differences in soil characteristics were observed among various tillage and fertilization regimes. Specifically, the soil pH in no-tillage and fertilization treatments was significantly lower than that in the conventional treatments. This reduction in pH under no-tillage may be attributed to the accumulation of organic matter in the topsoil, which increases ion concentrations and subsequently decreases pH levels [45].

Application of nitrogen fertilizers can lower soil pH through the nitrification process, where nitrogen is converted into pH-reducing compounds. Conversely, substituting inorganic fertilizers with organic fertilizers can mitigate soil acidification, as organic amendments often possess properties that help balance soil pH. In our study, the application of organic fertilizers resulted in lower soil pH, likely due to their pH-balancing effects, which helped neutralize the acidity in the locally alkaline soils.

Our findings indicated significant variations in soil properties among different long-term tillage and fertilization practices. Notably, the contents of organic matter (OM), total nitrogen (TN), total phosphorus (TP), ammonium nitrogen (AN), available phosphorus (AP), and available potassium (AK) in the NTM treatment were significantly higher than those in the C treatment (*p* < 0.05). The TP, AN, AP, and AK levels in manure treatments (TM and NTM) were higher than those in no-tillage (NT) and conventional tillage (T) treatments (*p* < 0.05), corroborating the findings of Manyiwa and Dikinya [46]. These results suggested that no-tillage practices enhanced soil nutrient content and availability, potentially due to improved soil water retention and aggregate stability. The choice of fertilizer type also plays a crucial role in determining soil nutrient content. Previous studies have shown that soil microbial diversity was positively associated with increases in soil organic carbon and total nitrogen while negatively correlated with soil pH under no-till conditions [47].

### 3.3. Effects of Tillage and Fertilization Management on Soil Fungal Diversity

Tillage practices not only modify soil properties but also alter the structure of soil microbial communities [19]. Dong et al. [48] demonstrated that no-tillage typically induces shifts in soil microbial communities, resulting in increases in microbial biomass and diversity. Conversely, long-term application of chemical fertilizers often inhibits the growth and development of key microbial groups. The supplementary application of organic fertilizers, on the other hand, can enhance microbial activity, accelerate nutrient turnover, and improve the richness of these critical groups [24,49,50]. Long-term fertilization of agricultural soils in Northern China with organic manure has been shown to produce distinct microbial community structures characterized by higher richness and diversity [51]. In our study, the manure treatments (NTM and TM) increased the number of operational taxonomic units (OTUs) by 8.39% to 15.95% compared to the fertilizer treatments (NT and T), while the fertilizer treatment (T) resulted in a 19.07% decrease in OTUs compared to the control (C). This suggests that the application of manure mitigates the adverse effects of fertilizers on OTU loss. Furthermore, no-tillage treatments (NT and NTM) enhanced OTUs by 8.39% to 15.95% compared to conventional tillage, with the highest number of OTUs observed in the NTM treatment and the lowest in the T treatment. Notably, soil fungal OTU richness was greater in no-tillage systems than in the conventional tillage systems, indicating a more passive and active role in agricultural ecosystem functions [52,53].

The Chao1 index further revealed that the no-tillage treatments exhibited higher fungal diversity than the conventional tillage systems [29,54]. Our studies showed tillage had significant effects on soil fungal Chao1 and Shannon index, and fertilizer type did not significantly affect soil fungal richness and diversities. There were no significant differences in Simpson and ACE indexes among treatments. The T and NT treatments had higher evenness, the NTM and TM treatments had lower evenness and the TM and C treatments had no obvious evenness differences (Table 3). The reduced disturbance associated with no-tillage allowed for crop residues to decompose into soil organic matter (SOM), which accumulated over time, thereby enhancing soil structure. The improved structure fostered the protection of fungal hyphae and specialized microbial niches, promoting the proliferation of soil microorganisms and their activities. Consequently, no-tillage systems were associated with greater microbial abundance compared to conventional tillage practices [50,55,56]. Overall, both chemical and organic fertilizers contributed to increased soil nutrient levels and enriched soil fungal diversity [57,58,59]. Morugán-Coronado et al. [38] showed organic fertilization contributed to a significant increase in fungal abundance, while lack of fertilization did not significantly affect fungal abundance. Wu et al. [60] found that organic materials enhanced fungal abundances, with more pronounced effects on alpha diversity in the fungal community compared to the bacterial community. Additionally, Bebber and Richards [61] noted that manure had a more substantial impact on functional and prokaryotic taxonomic diversity than chemical fertilizers, although no differences were observed in taxonomic diversity between fertilized and unfertilized fungal communities [62]. Our results indicated that the NTM treatment significantly increased fungal richness and diversity through appropriate tillage practices, and organic fertilizers further enhanced these parameters compared to chemical fertilizers.

### 3.4. Effects of Tillage and Fertilization Management on Soil Fungal Compositions

No-tillage [63] and organic manure application [64] increased soil aeration and oxygen concentrations and altered soil fungal community composition. The dominant phyla were *Ascomycota* (81.36%–90.24%), *Mucoromycota* (5.40%–12.83%), and *Basidiomycetes* (1.50%–8.53%) in the five treatments. There was no significant difference in *Ascomycetes,* but a significant difference appeared in *Mucorales* and *basidiomycetes* among treatments (*p* < 0.05) (Figure 3). This is generally consistent with the findings of Wang et al. [65]. Degrune et al. [66] and Zhou et al. [33] showed that *Ascomycota* was the most abundant phylum of all treated samples and was less affected by conservation tillage as a dominant group. However, *Ascomycetes* responded significantly to the supplementary manure application [67]. *Ascomycetes* have a competitive advantage in competing for niche space and resources, highlighting that *Ascomycetes* are important fungal decomposers in farmland ecosystems [68,69]. Therefore, the NTM treatment promoted the richness and diversity of fungal community composition.

At the genus level, we compared and analyzed the six key dominant genera: *Fusarium, Chaetomium, Humicola, Mortierella, Dendrostilbella,* and *Alternaria* (Figure 5). With the exception of *Humicola*, the other genera (*Fusarium, Chaetomium, Mortierella, Dendrostilbella,* and *Alternaria*) exhibited significant differences among the treatments (*p* < 0.05). These differences in soil fungal communities were influenced by tillage and fertilization practices. Notably, the abundance of *Humicola*, *Mortierella,* and *Dendrostilbella* in the NTM treatment was significantly higher than that in the other treatments, whereas *Fusarium, Chaetomium,* and *Alternaria* were significantly lower.

The long-term incorporation of straw and application of manure resulted in the accumulation of substantial amounts of residual organic matter, crop residue, humus, and pathogens in the topsoil. These conditions produced a conducive environment that served as potential inoculation sources, thereby increasing the risk of soil-borne diseases [70,71]. The genus *Mortierella*, as a beneficial microbe that dissolves phosphorus in soil [72] and coexists with photosynthetic organisms [73], was enriched in the soil, possibly because manure provided energy to photosynthetic organisms, thereby affecting the relative abundance of *Mortierella*.

Hierarchical clustering and PCoA analyses revealed distinct similarities in soil fungal communities under different tillage and fertilization treatments. Specifically, the C and T treatments clustered together, forming a unique group that clearly differed from the NT, TM, and NTM treatments. These findings support our hypothesis that NTM treatment enhances fungal community diversity. The observed shifts in fungal composition and diversity can be attributed to several factors. First, the increase in soil oxygen content due to conservation tillage and the incorporation of manure enhances soil pore connectivity. This improvement in aeration elevates oxygen concentrations, thereby promoting the abundance of aerobic fungal species and subsequently increasing the overall composition and diversity of the fungal community [63,64]. Second, on-till provides a suitable humidity environment for fungi, which promotes fungal abundance [74]. It may also accelerate the decomposition of crop residues, promote the increase of soil organic carbon content, and provide nutrient conditions for microbial absorption and utilization, resulting in different compositions of soil fungal communities. Third, long-term manure application improves nutrient availability by increasing microbial community diversity and species richness [34].

### 3.5. Soil Fungi Association Analysis with Soil Environmental Factors, Yield, and NUE

Soil microbial communities are closely linked to soil environmental factors. Changes in soil physical and chemical properties significantly influence the survival and reproduction of soil microbes. The activities of these microorganisms, in turn, facilitate soil nutrient cycling and material flow and underscore the importance of their interaction in improving soil quality. Redundancy analysis (RDA) revealed distinct correlations among dominant fungal communities and soil properties. Specifically, *Ascomycota* and *Zoopagomycota* both exhibited relatively high abundance and showed positive correlations with OM, AN, AP, TP, and AK. Conversely, *Basidiomycota* and *Chytidiomycota* negatively correlated with soil pH, while *Blastocladiomycota* and *Mucoromycota* exhibited no significant correlations with soil properties. The composition of fungal communities was notably influenced by soil properties across different treatments. As a dominant phylum in the rhizosphere, *Ascomycota* is closely associated with soil nitrogen. *Pleosporales* (*Ascomycota; Dothideomycetes*), which can directly utilize carbon resources from root exudates, often emerge as the predominant order within this phylum, thereby enhancing its overall dominance [75]. *Mucoromycota*, characterized by its sterile mycelium, typically represents the intracellular microbiota of early-differentiated fungi [76]. Furthermore, Bodenhausen et al. [77] demonstrated that *Mortierella* species were root endophytic fungi, and their community composition was affected by plant phosphorus content, which might be caused by the phosphorus content in straw incorporation and fertilizers used in experimental studies. *Basidiomycota* showed a strong ability to decompose lignin and lignocellulose complexes [78], which might be caused by the fact that no-tillage and straw returning provided the basis for anaerobic and high-lignin-content and anaerobic environments that increased pH values, which was beneficial to the growth and development of *Basidiomycota* and the increase of its abundance. Our study demonstrated that fungal community compositions correlated positively with *Ascomycota* and *Zoopagomycota* in the NTM and TM treatments and negatively correlated with Basidiomycota and Chytiomycotta in the C, T, and NT treatments. Organic manure provided the necessary nutrients for microorganisms and promoted the growth of *Ascomycota* and *Zoopagomycota*. In contrast, the growth of *Basidiomycota* and *Chytiomycotta* was inhibited in C, T, and NT treatments without organic fertilizer.

*Mortierella* has been described as beneficial soil microorganisms that can be used to improve soil micro-ecology and promote plant growth [79]. Our results supported this finding as the relative abundance of *Mortierella* was positively correlated with wheat yield (Figure 9). This was consistent with the results obtained in grape [80]. *Mortierella* was positively correlated with NUE. *Fusarium* is a widespread pathogenic fungus that can cause a variety of plant diseases, reducing crop yield [81,82]. The relative abundance of *Fusarium* in NTM was the lowest and was negatively correlated with wheat yield, AE_N_, PFP_N_, apparent RE_N,_ and accumulated RE_N_. *Alternaria* has toxigenic species that produce mycotoxins, such as tenuazonic acid and alterotoxins, and decrease crop yield [83,84]. The relative abundance of *Alternaria* was the lowest in the NTM treatment and showed significant negative correlations with wheat yield, agronomic efficiency of nitrogen (AE_N_), partial factor productivity of nitrogen (PFP_N_), apparent recovery efficiency of nitrogen (RE_N_), and accumulated RE_N_ (*p* < 0.05) (Figure 9). These findings suggest that long-term tillage and the application of manure may decrease the risk of disease infections in wheat.

## 4. Materials and Methods

### 4.1. Site Description

The long-term field tillage and fertilization experiment was initiated in 2008 at the Niujiawa Agricultural Experimental Farm, located in Yuncheng City, Shanxi Province, China (35°11′ N, 111°05′ E). The cropping system employed is a wheat-summer fallow rotation within a dryland agricultural context. The site has a temperate continental monsoon climate, with an average annual temperature of 13.2 °C, an annual sunshine duration of 2293 h, a frost-free period lasting 212 days, and a total annual precipitation of 525 mm. In the wheat-growing season, the precipitation was 542.8 mm from July 2020 to May 2021. The experimental site has cinnamon soil, specifically silty clay loam (composed of 17.5% clay, 28.0% sandy soil, and 54.5% silty sand) texture. Before the experiment, the following chemical and physical properties of the topsoil layer (0–20 cm) had a pH of 8.15, an organic matter content of 10.6 g/kg, total nitrogen content of 0.89 g/kg, a total phosphorus content of 1.08 g/kg, an available nitrogen content of 56.9 mg/kg, an available phosphorus content of 13.1 mg/kg, and an available potassium content of 159.6 mg/kg.

### 4.2. Experimental Design and Sampling

The long-term experiment used a randomized block design with five treatments: (1) C, conventional tillage without any fertilizer; (2) NT, no-tillage with chemical fertilizer; (3) NTM, no-tillage with chemical fertilizer and manure; (4) T, conventional tillage with chemical fertilizer; and (5) TM, conventional tillage with chemical fertilizer and manure. Each treatment had three repetitions, and the plot size was 60 m^2^. Straw stubble retaining was about 15 cm high after wheat harvest, and straw was manually crushed in all treatments. For conventional tillage, the soil was plowed annually to a depth of about 25 cm in mid-July, and rotary tillage (to a depth of about 15 cm) was conducted to prepare the soil and incorporate fertilizers before sowing. For no-tillage treatment operation, the straw was crushed and covered on the soil surface between June and September. Fertilizers and rotary tillage were used when mechanical sowing was applied. Except for treatment C, chemical fertilizers were applied to the other plots with 180 kg-N/ha and 150 kg-P_2_O_5_/ha. The chemical N and P fertilizers were urea (N: 46%) and calcium superphosphate (P_2_O_5_: 18%), which were applied as basal fertilizers before sowing. For the NTM and TM treatments, decomposed chicken manure was applied at a rate of 1500 kg/ha before sowing. The manure was decomposed chicken manure, which contained 24.98% organic matter, 19.9 g/kg of total N, 30.2 g/kg of total P, and 20.7 g/kg of total K. Wheat was mechanically sown in early October and harvested in early June. Follow was practiced between June and September. In recent years, the rainfed wheat cultivar Yunhan 618 was used. The management practices were consistent during the experiment.

### 4.3. Sampling and Measurements

Grain yield, adjusted to a moisture content of 14%, was determined by harvesting three 6 m^2^ sections from the center of each plot. Soil samples were collected immediately following the wheat harvest in 2021. Ten soil cores were extracted from each plot at a depth of 0–20 cm and thoroughly mixed to produce a homogeneous sample. Any debris (e.g., gravel and roots) was removed from each plot, and the samples were placed into sterile sealing bags, stored in an ice box, and transported to the laboratory. A portion of the samples was preserved at −80 °C for the analysis of soil fungal communities, while the other portion was air-dried for the determination of soil physicochemical properties.

Wheat samples were divided into grain and straw and subsequently dried at 70 °C. Total nitrogen (N) concentration was analyzed by Kjeldahl method using a SKD-800 automatic analyzer (Shanghai Peiou Analytical Instrument Co., Ltd., Shanghai, China). Soil organic matter (OM) was detemined by the potassium dichromate oxidation-photo-colorimetric method using a spectrophotometer ( Shanghai Metash Instruments Co., Ltd., Shanghai, China). Available nitrogen (AN) was determined using the alkali-diffusion method. Total phosphorus (TP) was quantified using the HClO_4_-H_2_SO_4_ method, and available phosphorus (AP) was assessed through sodium bicarbonate extraction Mo-Sb colorimetry (Olsen method). Available potassium (AK) was measured by the ammonium acetate extraction flame photometric method using an AP1302 flame spectrophotometer (Shanghai Instruments Group Co., Ltd., Shanghai, China). Soil pH was measured by a FE20 pH meter (Mettler Toledo, Shanghai, China). 

Four indexes were calculated to investigate the N use efficiency (NUE) as follows [11,85]:

Apparent recovery efficiency of N (Apparent RE_N_, %) = (N_uptake_ − 0 N_uptake_)/N_rate_ × 100% (1)

Accumulated recovery efficiency of N (Accumulated RE_N_) = (accumulated N_uptake_ − accumulated 0 N_uptake_)/accumulated N_rate_ × 100% (2)

Agronomic efficiency of N (AE_N_, kg/kg) = (N_yield_ − 0 N_yield_)/N_rate_ (3)

Partial factor productivity of N (PFP_N_, kg/kg) = N_yield_/N_rate_ (4)

where N_uptake_, and 0 N_uptake_, indicate N_uptake_ from N, and 0 treatment plots, respectively, N_yield_ represent grain yield in the N application treatments, and 0 N_yield_ represent grain yield in the control plots. The N_rate_ is the total amount of N applied during wheat growth.

### 4.4. DNA Extraction, PCR Amplification, and Sequencing

Total soil DNA was extracted from freeze-dried soil samples (0.5 g) using a Power Soil DNA Isolation Kit (Omega Bio-tek, Norcross, GA, USA). The concentration and purity of the extracts were assessed using a Nanodrop ND-1000 nucleic acid (Nanodrop Technologies, Wilmington, DE, USA) quantifier and 1% agarose gel electrophoresis. DNA samples were stored at −20 °C. The ITS gene of soil fungi was amplified using the primers ITS1F (5′-CTTGGTCATTTAGAGGATAA-3′) and ITS2R (5′-GCTGTGTTCATCGATGC-3′).

The PCR reaction system (25 μL) included: 5× reaction buffer (5 μL), 5× GC buffer (5 μL), dNTP (2.5 mmol/L, 2 μL), forward primers (10 μmol/L, 1 μL), reverse primers (10 μmol/L, 1 μL), DNA template (2 μL), ddH_2_O (8.75 μL), and Q5 DNA polymerase (0.25 μL). The PCR conditions consisted of 30 cycles, which included an initial denaturation at 98 °C for 2 min, denaturation at 98 °C for 15 s, annealing at 55 °C for 30 s, extension at 72 °C for 30 s, and a final extension at 72 °C for 5 min. After PCR amplification, the products were subjected to 2% agarose gel electrophoresis. Required bands were excised and purified using a Pure Link rapid gel extraction kit, and the amplicon was sequenced on the Illumina MiSeq platform. The QuantiFluor™-ST blue fluorescence quantitative system was used to detect and quantify the PCR products, which were then mixed according to the required proportions for sequencing. Sequences were clustered into several operational taxonomic units (OTUs) using QIIME software, version 1.9.0 and the OTUs of each soil sample were calculated. Comparisons of sequences and species classification were conducted using the UNITE’s fungal database (Release 8.2, http://unite.ut.ee/index.php accessed on 9 October 2021). OTU sequences with a similarity level of 97% were analyzed for bioinformatics taxonomy using the RDP classifier Bayesian algorithm, and community composition was assessed at both the phylum and genus levels. Soil species richness, Shannon–Wiener index, and Chao1 index were also calculated (sequencing company: Shanghai Biozeron Technology Co., Ltd., Shanghai, China).

We conducted a one-way analysis of variance (ANOVA) and *t*-test to analyze the experimental data by using IBM Statistics SPSS 20.0 (International Business Machines Corp, New York, NY, USA). The LSD method was used to determine the significance level with an α of 0.05. After sequencing, OTU clustering was performed for effective sequences with similarity ≥ 97%. The index evaluation was performed by using Mothur (version v.1.30.1) to calculate the Chao l index, Shannon index, Simpson index, ACE index, and evenness index of fungi, and the significance test was conducted to evaluate the differences between treatments. The Venn diagram, relative abundance histogram, and cluster heat map of sample species composition were drawn using the R software version 3.6.3. PCoA and Redundancy Analysis (RDA) were analyzed and plotted using it. The rest of the bar charts used Origin (v.2021) (Origin Lab Corp, Northampton, MA, USA). LEfSe used linear discriminant analysis (LDA) to estimate the impact of each component (species) abundance on the difference effect. The unweighted pair-group method with the arithmetic mean (UPGMA) algorithm was used to construct a tree structure, and the tree relationship form was obtained for visualization analysis.

## 5. Conclusions

No-tillage practices and manure application not only significantly enhanced soil fertility but also altered the structure and composition of the soil fungal community. These measures improved fungal richness and diversity, leading to increased wheat yield and nitrogen use efficiency. Specifically, *Mortierella* and *Dendrostilbella* showed significant positive correlations with wheat yield, apparent nitrogen efficiency (AE_N_), partial factor productivity of nitrogen (PFP_N_), apparent recovery efficiency of nitrogen (RE_N_), and accumulated RE_N_. In contrast, *Fusarium*, *Chaetomium*, and *Alternaria* exhibited significant negative correlations with these metrics (*p* < 0.05). The findings from this study provide a theoretical foundation for improving soil fertility and enhancing the role of fungal communities in agricultural ecosystems.

## Figures and Tables

**Table 1:** Wheat grain yield, nitrogen agronomic efficiency (AE_N_), partial factor productivity (PEP_N_), apparent recovery efficiency (Apparent RE_N_), and accumulated recovery efficiency (Accumulated RE_N_) in the treatments.

Treatments	Grain Yield (kg/ha)	AE_N_ (kg/kg)	PEP_N_ (kg/kg)	Apparent RE_N_ (%)	Accumulated RE_N_ (%)
C	1767.00 ± 55.90 c	-	-	-	-
NT	2931.17 ± 564.76 b	6.47 ± 3.12 b	16.28 ±3.14 b	23.69 ± 9.43 b	46.85 ±10.22 bc
NTM	3974.78 ± 165.60 a	12.27 ± 1.13 a	22.08 ± 0.92 a	45.90 ± 3.52 a	64.17 ± 2.39 a
T	2504.65 ± 118.67 b	4.10 ± 0.82 b	13.91 ± 0.66 b	15.27 ± 2.35 b	37.29 ± 4.22 c
TM	2985.62 ± 222.34 b	6.77 ±1.06 b	16.59 ± 1.24 b	25.44 ± 3.33 b	49.76 ± 2.86 b

Note: The numeric values represent mean ± SD (standard deviation). Different lowercase letters in a column indicate significant differences among treatments (*p* < 0.05), respectively.

**Table 2:** Soil properties under different tillage and fertilization management.

Treatment	OM (g/kg)	TN (g/kg)	TP (g/kg)	AN (mg/kg)	AP (mg/kg)	AK (mg/kg)	pH
C	18.15 ± 1.37 c	1.59 ± 0.16 a	1.50 ± 0.06 d	70.00 ± 7.00 c	15.04 ± 1.67 d	259.27 ± 2.79 c	8.19 ± 0.02 a
NT	22.64 ± 0.78 b	0.93 ± 0.16 c	2.10 ± 0.13 c	88.67 ± 10.69 bc	40.03 ± 5.36 c	228.55 ± 1.34 e	8.07 ± 0.02 c
NTM	30.98 ± 1.45 a	1.73 ± 0.09 a	4.68 ± 0.48 a	133.00 ± 14.00 a	130.69 ± 18.33 a	490.05 ± 7.87 a	8.00 ± 0.01 d
T	21.29 ± 1.02 b	1.59 ± 0.16 a	1.66 ± 0.18 d	105.00 ± 14.00 b	36.71 ± 0.77 c	247.75 ± 5.42 d	8.12 ± 0.01 b
TM	22.94 ± 0.18 b	1.31 ± 0.16 b	3.00 ± 0.17 b	128.33 ± 10.69 a	115.66 ± 2.13 b	335.08 ± 4.31 b	8.06 ± 0.02 c
ANOVA							
Tillage	58.464 **	8.514 *	157.842 **	12.195 **	142.463 **	1404.910 **	185.606 **
Fertilizer	99.431 **	14.080 **	77.378 **	30.071 **	171.685 **	1259.080 **	75.884 **
Tillage × Fertilizer	29.786 **	21.226 **	70.929 **	0.488	47.916 **	2339.443 **	0.152

Note: The numeric values represent mean ± SD (standard deviation). OM: organic matter; TN: total nitrogen; TP: total phosphorus; AN: available nitrogen; AP: available phosphorus; AK: available potassium. Different lowercase letters in a column indicate significant differences at *p* < 0.05. * and ** show significant levels at 5% and 1%.

**Table 3:** Alpha diversity indices of fungal community under five treatments.

Treatment	Chaol Index	Shannon Index	Simpson Index	ACE Index	Evenness Index
C	330.54 ± 15.79 ab	5.00 ± 0.29 c	0.04 ± 0.00 a	351.43 ± 23.28 a	0.60 ± 0.03 b
NT	342.13 ± 21.36 ab	5.89 ± 0.31 ab	0.03 ± 0.01 a	337.90 ± 39.75 a	0.72 ± 0.03 a
NTM	354.20 ± 19.55 a	5.98 ± 0.09 a	0.08 ± 0.01 a	347.31 ± 30.81 a	0.71 ± 0.02 a
T	291.69 ± 41.53 b	5.81 ± 0.18 ab	0.04 ± 0.01 a	294.40 ± 39.54 a	0.72 ± 0.04 a
TM	352.52 ± 34.93 a	5.20 ± 0.67 bc	0.09 ± 0.06 a	327.90 ± 21.12 a	0.63 ± 0.09 ab
ANOVA					
Tillage	5.106 *	6.399 *	1.070	1.145	3.848
Fertilizer	2.459	2.570	3.488	0.861	2.801
Tillage × Fertilizer	2.412	3.031	2.204	3.258	4.876

The results are shown as the mean ± SD (standard deviation). Values with different lowercase letters are significantly different at the 0.05 level. * indicates a significant level at 5%.

## Data Availability

Data are contained within the article.
